# [1,4-Bis(diphenyl­phosphan­yl)butane-κ^2^ *P*,*P*′]chlorido(η^5^-inden­yl)ruthenium(II)

**DOI:** 10.1107/S1600536811015121

**Published:** 2011-04-29

**Authors:** Hui-Ling Sung, Tze-Min Her, Wen-Hsien Su, Chin-Pao Cheng

**Affiliations:** a Department of Mathematics and Science (Pre-college), National Taiwan Normal University, Taiwan; b Department of Chemical and Materials Engineering, Lunghwa University of Science and Technology, Taiwan; c Department of Mechatronic Technology, National Taiwan Normal University, Taiwan

## Abstract

Facile ligand substitution is observed when the ruthenium chloride complex [Ru(η^5^-C_9_H_7_)Cl(PPh_3_)_2_] is treated with 1,4-bis­(diphenyl­phosphan­yl)butane in refluxing toluene yielding the title compound, [Ru(C_9_H_7_)Cl(C_28_H_28_P_2_)]. The Ru^II^ atom has a typical piano-stool coordination, defined by the indenyl ligand, one Cl atom and two phosphanyl P atoms. The Ru—P bond lengths are 2.2502 (9) and 2.2968 (8) Å.

## Related literature

For general background to the title compound and other [Ru(η^5^-C_9_H_7_)Cl(diphos)] compounds, see: Oro *et al.* (1985 ▶); Tanase *et al.* (1994 ▶). For the chemistry of [Ru(η^5^-C_9_H_7_)Cl(diphos)], see: Franco (1989 ▶).
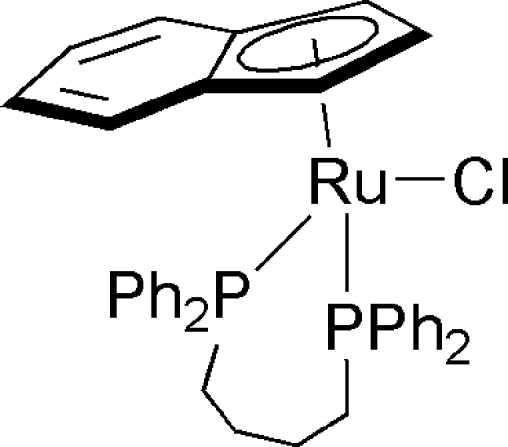


## Experimental

### 

#### Crystal data

[Ru(C_9_H_7_)Cl(C_28_H_28_P_2_)]
*M* *_r_* = 678.11
Monoclinic, 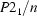

*a* = 12.6567 (2) Å
*b* = 15.7502 (3) Å
*c* = 15.9419 (3) Å
β = 103.165 (1)°
*V* = 3094.42 (10) Å^3^
*Z* = 4
Mo *K*α radiation
μ = 0.72 mm^−1^
*T* = 200 K
0.55 × 0.48 × 0.38 mm

#### Data collection

Nonius KappaCCD diffractometer
Absorption correction: multi-scan (*SADABS*; Bruker, 2005 ▶) *T*_min_ = 0.704, *T*_max_ = 0.821
18461 measured reflections
5443 independent reflections
4355 reflections with *I* > 2σ(*I*)
*R*_int_ = 0.060

#### Refinement

*R*[*F* ^2^ > 2σ(*F* ^2^)] = 0.035
*wR*(*F* ^2^) = 0.102
*S* = 0.91
5443 reflections
370 parameters
H-atom parameters constrained
Δρ_max_ = 0.36 e Å^−3^
Δρ_min_ = −0.71 e Å^−3^

### 

Data collection: *COLLECT* (Nonius, 1998 ▶); cell refinement: *DENZO*/*SCALEPACK* (Otwinowski & Minor, 1997 ▶); data reduction: *DENZO*/*SCALEPACK*; program(s) used to solve structure: *SHELXS97* (Sheldrick, 2008 ▶); program(s) used to refine structure: *SHELXL97* (Sheldrick, 2008 ▶); molecular graphics: *ORTEP-3 for Windows* (Farrugia, 1997 ▶); software used to prepare material for publication: *WinGX* (Farrugia, 1999 ▶).

## Supplementary Material

Crystal structure: contains datablocks global, I. DOI: 10.1107/S1600536811015121/bv2178sup1.cif

Structure factors: contains datablocks I. DOI: 10.1107/S1600536811015121/bv2178Isup2.hkl

Additional supplementary materials:  crystallographic information; 3D view; checkCIF report

## Figures and Tables

**Fig. 1.**
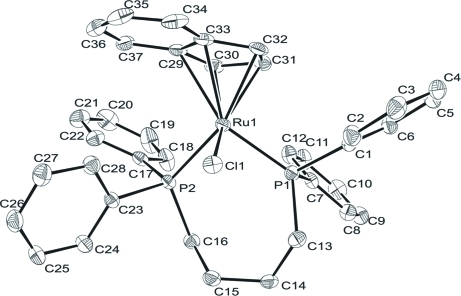
View of the title compound showing displacement ellipsoids at the 30% probability level. H atom are omitted for clarity.

## supplementary crystallographic information

### Comment

Reaction of [Ru(η^5^-C_9_H_7_)Cl(diphos)] with phenylacetylene in the presence of alcoholic KOH yielded ruthenium acetylide complexes [Ru(η^5^-C_9_H_7_)(η^1^-CCPh)((diphos)] (diphos =1,2-bis(diphenylphosphanyl)butane (dppe), 1,2-bis(diphenylphosphanyl)butane (dppp), 1,2-bis(diphenylphosphanyl)butane (dppb)) in good yield (Tanase *et al.*, 1994). Treatment of the complex [Ru(η^5^-C_9_H_7_)Cl(PPh_3_)_2_] with 1,2-bis(diphenylphosphanyl)butane in toluene afforded the title compound [Ru(η^5^-C_9_H_7_)Cl(dppb)] (Figure 1). In the crystal structure of the title compound, the bidentate ligand dppb is coordinated to Ru with an P—Ru—P angle of 94.88 (3)°. The Ru—P bond lengths are 2.2502 (9) and 2.2968 (8) Å, respectively.

### Experimental

The title compound was prepared by a similar method used for the dppe ligand in the previous literature procedure of Oro, *et al.* (1985) The red-brown crystals of the title compound for X-ray structure analysis were obtained by slow diffusion of diethyl ether into a CH_2_Cl_2_ solution at room temperature for 3 days. Spectroscopic analysis: ^1^H NMR (CDCl_3_, 298 K, δ, p.p.m.): 7.48 — 7.13 (m, 24H, 20H of Ph and 4H of indenyl group); 4.49 (br, 1H of indenyl group); 3.82 (br, 2H of indenyl group); 3.21, 2.03, 1.80, 1.52 (m, 2H each one, CH_2_ of dppb). ^31^P{^1^H} NMR (CDCl_3_, 298 K, δ, p.p.m.): 49.5. ^13^C{^1^H} NMR (CDCl_3_, 298 K, δ, p.p.m.): 137.0 —128.4 (Ph); 122.6 (C-5, 6); 121.5 (C-4, 7); 115.7 (C-2); 99.7 (C-1, 3), 31.8 (t, two PCH_2_ of dppb, J_P—C_= 1.30 Hz); 27.4 (s, two CH_2_ of dppb). HRMS (ESI, m/*z*): 678.2 (*M*^+^). Anal. Calcd for C_37_H_35_ P_2_ClRu: C: 65.53, H: 5.20, Found: C: 65.58, H: 5.23.

### Refinement

All H atoms were initially located in a difference map, but were constrained to an idealized geometry. Constrained bond lengths and isotropic displacement parameters: C—H = 0.95 Å and *U*_iso_(H) = 1.2 *U*_eq_(C) for aromatic H atoms, and C—H = 0.99 Å and *U*_iso_(H) = 1.2 *U*_eq_(C) for methylene.

### Crystal data

**Table:**

[Ru(C_9_H_7_)Cl(C_28_H_28_P_2_)]	*F*(000) = 1392
*M**_r_* = 678.11	*D*_x_ = 1.456 Mg m^−^^3^
Monoclinic, *P*2_1_/*n*	Mo *K*α radiation, λ = 0.71073 Å
Hall symbol: -P 2yn	Cell parameters from 13485 reflections
*a* = 12.6567 (2) Å	θ = 2.0–25.0°
*b* = 15.7502 (3) Å	µ = 0.72 mm^−^^1^
*c* = 15.9419 (3) Å	*T* = 200 K
β = 103.165 (1)°	Prism, red–brown
*V* = 3094.42 (10) Å^3^	0.55 × 0.48 × 0.38 mm
*Z* = 4	

### Data collection

**Table:**

Nonius KappaCCD diffractometer	5443 independent reflections
Radiation source: fine-focus sealed tube	4355 reflections with *I* > 2σ(*I*)
graphite	*R*_int_ = 0.060
Detector resolution: 9 pixels mm^-1^	θ_max_ = 25.0°, θ_min_ = 2.9°
CCD rotation images, thick slices scans	*h* = −14→15
Absorption correction: multi-scan (*SADABS*; Bruker, 2005)	*k* = −18→18
*T*_min_ = 0.704, *T*_max_ = 0.821	*l* = −18→18
18461 measured reflections	

### Refinement

**Table:**

Refinement on *F*^2^	Primary atom site location: structure-invariant direct methods
Least-squares matrix: full	Secondary atom site location: difference Fourier map
*R*[*F*^2^ > 2σ(*F*^2^)] = 0.035	Hydrogen site location: inferred from neighbouring sites
*wR*(*F*^2^) = 0.102	H-atom parameters constrained
*S* = 0.91	*w* = 1/[σ^2^(*F*_o_^2^) + (0.0707*P*)^2^ + 0.5716*P*] where *P* = (*F*_o_^2^ + 2*F*_c_^2^)/3
5443 reflections	(Δ/σ)_max_ < 0.001
370 parameters	Δρ_max_ = 0.36 e Å^−^^3^
0 restraints	Δρ_min_ = −0.71 e Å^−^^3^

### Special details

**Table:**

Geometry. All e.s.d.'s (except the e.s.d. in the dihedral angle between two l.s. planes) are estimated using the full covariance matrix. The cell e.s.d.'s are taken into account individually in the estimation of e.s.d.'s in distances, angles and torsion angles; correlations between e.s.d.'s in cell parameters are only used when they are defined by crystal symmetry. An approximate (isotropic) treatment of cell e.s.d.'s is used for estimating e.s.d.'s involving l.s. planes.
Refinement. Refinement of *F*^2^ against ALL reflections. The weighted *R*-factor *wR* and goodness of fit *S* are based on *F*^2^, conventional *R*-factors *R* are based on *F*, with *F* set to zero for negative *F*^2^. The threshold expression of *F*^2^ > σ(*F*^2^) is used only for calculating *R*-factors(gt) *etc*. and is not relevant to the choice of reflections for refinement. *R*-factors based on *F*^2^ are statistically about twice as large as those based on *F*, and *R*-factors based on ALL data will be even larger.

### Fractional atomic coordinates and isotropic or equivalent isotropic displacement parameters (Å^2^)

**Table:**

	*x*	*y*	*z*	*U*_iso_*/*U*_eq_	
C1	0.9824 (3)	0.0561 (2)	0.6534 (2)	0.0310 (7)	
C2	1.0539 (3)	0.0051 (2)	0.7111 (2)	0.0416 (9)	
H2	1.0262	−0.0376	0.7423	0.050*	
C3	1.1652 (3)	0.0158 (3)	0.7235 (2)	0.0492 (10)	
H3	1.2134	−0.0188	0.7638	0.059*	
C4	1.2059 (3)	0.0773 (3)	0.6769 (3)	0.0485 (10)	
H4	1.2821	0.0844	0.6849	0.058*	
C5	1.1364 (3)	0.1276 (2)	0.6196 (3)	0.0457 (9)	
H5	1.1644	0.1698	0.5881	0.055*	
C6	1.0246 (3)	0.1170 (2)	0.6073 (2)	0.0375 (8)	
H6	0.9768	0.1518	0.5669	0.045*	
C7	0.7758 (2)	0.1115 (2)	0.5546 (2)	0.0308 (7)	
C8	0.7854 (3)	0.0919 (2)	0.4711 (2)	0.0367 (8)	
H8	0.8231	0.0420	0.4612	0.044*	
C9	0.7405 (3)	0.1445 (2)	0.4029 (2)	0.0415 (9)	
H9	0.7477	0.1309	0.3464	0.050*	
C10	0.6851 (3)	0.2169 (2)	0.4166 (3)	0.0478 (9)	
H10	0.6539	0.2529	0.3696	0.057*	
C11	0.6753 (3)	0.2367 (2)	0.4985 (2)	0.0470 (9)	
H11	0.6376	0.2866	0.5081	0.056*	
C12	0.7198 (3)	0.1845 (2)	0.5667 (2)	0.0392 (8)	
H12	0.7120	0.1987	0.6229	0.047*	
C13	0.8163 (3)	−0.06514 (19)	0.6006 (2)	0.0344 (8)	
H13A	0.8628	−0.0708	0.5587	0.041*	
H13B	0.8445	−0.1055	0.6481	0.041*	
C14	0.7023 (3)	−0.0944 (2)	0.5559 (2)	0.0373 (8)	
H14A	0.6736	−0.0546	0.5080	0.045*	
H14B	0.7084	−0.1508	0.5300	0.045*	
C15	0.6194 (3)	−0.1009 (2)	0.6115 (2)	0.0387 (8)	
H15A	0.6553	−0.1269	0.6673	0.046*	
H15B	0.5608	−0.1398	0.5829	0.046*	
C16	0.5680 (3)	−0.0169 (2)	0.6296 (2)	0.0333 (8)	
H16A	0.4890	−0.0258	0.6224	0.040*	
H16B	0.5781	0.0252	0.5859	0.040*	
C17	0.5251 (2)	0.1131 (2)	0.7400 (2)	0.0325 (8)	
C18	0.4995 (3)	0.1697 (2)	0.6714 (3)	0.0486 (10)	
H18	0.5268	0.1602	0.6214	0.058*	
C19	0.4349 (3)	0.2393 (3)	0.6752 (3)	0.0653 (13)	
H19	0.4189	0.2778	0.6282	0.078*	
C20	0.3931 (3)	0.2536 (3)	0.7471 (3)	0.0618 (12)	
H20	0.3500	0.3024	0.7499	0.074*	
C21	0.4144 (3)	0.1970 (3)	0.8142 (3)	0.0505 (10)	
H21	0.3844	0.2057	0.8631	0.061*	
C22	0.4798 (3)	0.1269 (2)	0.8105 (2)	0.0378 (8)	
H22	0.4939	0.0877	0.8569	0.045*	
C23	0.5811 (3)	−0.05033 (19)	0.8083 (2)	0.0288 (7)	
C24	0.4915 (3)	−0.1027 (2)	0.7832 (2)	0.0385 (8)	
H24	0.4519	−0.1021	0.7250	0.046*	
C25	0.4590 (3)	−0.1557 (2)	0.8414 (3)	0.0495 (10)	
H25	0.3979	−0.1917	0.8230	0.059*	
C26	0.5150 (3)	−0.1566 (2)	0.9261 (2)	0.0479 (10)	
H26	0.4920	−0.1928	0.9662	0.058*	
C27	0.6037 (3)	−0.1055 (2)	0.9528 (3)	0.0474 (9)	
H27	0.6418	−0.1058	1.0115	0.057*	
C28	0.6382 (3)	−0.0532 (2)	0.8944 (2)	0.0372 (8)	
H28	0.7011	−0.0191	0.9130	0.045*	
C29	0.7860 (3)	0.1711 (2)	0.8823 (2)	0.0338 (8)	
C30	0.7742 (3)	0.2060 (2)	0.7974 (2)	0.0371 (8)	
H30	0.7118	0.2411	0.7658	0.045*	
C31	0.8750 (3)	0.1951 (2)	0.7732 (3)	0.0432 (9)	
H31	0.8948	0.2215	0.7218	0.052*	
C32	0.9457 (3)	0.1465 (2)	0.8371 (2)	0.0440 (9)	
H32	1.0228	0.1317	0.8380	0.053*	
C33	0.8911 (3)	0.1335 (2)	0.9061 (2)	0.0368 (8)	
C34	0.9246 (3)	0.0927 (2)	0.9869 (3)	0.0490 (10)	
H34	0.9951	0.0686	1.0038	0.059*	
C35	0.8541 (4)	0.0886 (3)	1.0400 (3)	0.0541 (11)	
H35	0.8751	0.0594	1.0934	0.065*	
C36	0.7508 (3)	0.1267 (3)	1.0172 (2)	0.0551 (11)	
H36	0.7038	0.1234	1.0558	0.066*	
C37	0.7170 (3)	0.1684 (2)	0.9408 (2)	0.0412 (9)	
H37	0.6480	0.1953	0.9271	0.049*	
Ru1	0.800240 (19)	0.071649 (15)	0.775247 (16)	0.02673 (11)	
P1	0.83580 (7)	0.04314 (5)	0.64612 (5)	0.0283 (2)	
P2	0.62274 (6)	0.02764 (5)	0.73711 (5)	0.0266 (2)	
Cl1	0.85934 (7)	−0.07244 (5)	0.82062 (5)	0.0343 (2)	

### Atomic displacement parameters (Å^2^)

**Table:**

	*U*^11^	*U*^22^	*U*^33^	*U*^12^	*U*^13^	*U*^23^
C1	0.0251 (17)	0.0342 (18)	0.0342 (18)	0.0036 (14)	0.0077 (14)	−0.0051 (15)
C2	0.0318 (19)	0.052 (2)	0.042 (2)	0.0030 (17)	0.0106 (16)	0.0007 (18)
C3	0.035 (2)	0.066 (3)	0.045 (2)	0.014 (2)	0.0067 (17)	−0.005 (2)
C4	0.0260 (19)	0.061 (3)	0.061 (3)	−0.0005 (18)	0.0146 (18)	−0.021 (2)
C5	0.038 (2)	0.043 (2)	0.060 (2)	−0.0085 (18)	0.0201 (19)	−0.0107 (19)
C6	0.0314 (19)	0.0367 (19)	0.047 (2)	−0.0018 (15)	0.0137 (16)	−0.0041 (17)
C7	0.0242 (17)	0.0314 (18)	0.0376 (18)	−0.0022 (14)	0.0087 (14)	0.0053 (15)
C8	0.038 (2)	0.0350 (19)	0.037 (2)	−0.0002 (15)	0.0075 (16)	0.0020 (16)
C9	0.038 (2)	0.048 (2)	0.038 (2)	−0.0024 (17)	0.0059 (16)	0.0065 (17)
C10	0.042 (2)	0.049 (2)	0.050 (2)	0.0052 (19)	0.0044 (18)	0.0139 (19)
C11	0.041 (2)	0.045 (2)	0.058 (2)	0.0146 (17)	0.0168 (18)	0.0140 (19)
C12	0.0338 (19)	0.040 (2)	0.046 (2)	0.0034 (16)	0.0132 (16)	0.0079 (17)
C13	0.039 (2)	0.0301 (18)	0.0333 (19)	0.0057 (15)	0.0070 (15)	−0.0001 (15)
C14	0.049 (2)	0.0268 (17)	0.0370 (19)	−0.0065 (16)	0.0119 (17)	−0.0082 (15)
C15	0.048 (2)	0.0295 (17)	0.0388 (19)	−0.0092 (16)	0.0103 (17)	−0.0013 (16)
C16	0.0325 (18)	0.0366 (19)	0.0288 (17)	−0.0044 (15)	0.0024 (14)	0.0011 (15)
C17	0.0227 (17)	0.0271 (17)	0.046 (2)	−0.0022 (14)	0.0042 (15)	−0.0005 (16)
C18	0.038 (2)	0.042 (2)	0.070 (3)	0.0121 (17)	0.0211 (19)	0.020 (2)
C19	0.060 (3)	0.045 (2)	0.099 (4)	0.019 (2)	0.036 (3)	0.031 (2)
C20	0.044 (2)	0.041 (2)	0.100 (4)	0.0171 (19)	0.017 (2)	0.002 (2)
C21	0.041 (2)	0.051 (2)	0.059 (3)	0.0117 (19)	0.0079 (19)	−0.011 (2)
C22	0.0329 (19)	0.041 (2)	0.0368 (19)	0.0056 (16)	0.0019 (15)	−0.0053 (16)
C23	0.0267 (17)	0.0256 (16)	0.0351 (18)	0.0004 (13)	0.0089 (14)	0.0027 (14)
C24	0.0356 (19)	0.0381 (19)	0.042 (2)	−0.0059 (16)	0.0096 (16)	0.0016 (17)
C25	0.052 (2)	0.046 (2)	0.057 (3)	−0.0180 (19)	0.026 (2)	−0.0033 (19)
C26	0.060 (3)	0.042 (2)	0.049 (2)	−0.0032 (19)	0.028 (2)	0.0087 (18)
C27	0.054 (2)	0.049 (2)	0.042 (2)	0.004 (2)	0.0167 (19)	0.0056 (18)
C28	0.039 (2)	0.0377 (19)	0.0352 (19)	−0.0007 (16)	0.0086 (16)	0.0039 (15)
C29	0.0290 (18)	0.0273 (17)	0.043 (2)	−0.0038 (14)	0.0033 (15)	−0.0153 (15)
C30	0.0365 (19)	0.0246 (17)	0.048 (2)	0.0007 (15)	0.0049 (16)	−0.0097 (16)
C31	0.043 (2)	0.0296 (18)	0.061 (2)	−0.0099 (16)	0.0204 (19)	−0.0128 (18)
C32	0.0247 (18)	0.045 (2)	0.062 (2)	−0.0120 (16)	0.0086 (17)	−0.0266 (19)
C33	0.0263 (17)	0.0338 (18)	0.046 (2)	−0.0023 (15)	−0.0015 (15)	−0.0188 (16)
C34	0.050 (2)	0.038 (2)	0.049 (2)	0.0065 (18)	−0.0089 (19)	−0.0206 (18)
C35	0.067 (3)	0.053 (2)	0.034 (2)	0.004 (2)	−0.006 (2)	−0.0154 (18)
C36	0.058 (3)	0.068 (3)	0.040 (2)	−0.011 (2)	0.013 (2)	−0.021 (2)
C37	0.0307 (19)	0.051 (2)	0.042 (2)	−0.0026 (16)	0.0072 (16)	−0.0208 (18)
Ru1	0.02312 (16)	0.02491 (16)	0.03191 (17)	−0.00150 (10)	0.00574 (11)	−0.00442 (11)
P1	0.0259 (4)	0.0272 (4)	0.0325 (5)	0.0015 (3)	0.0077 (4)	−0.0010 (4)
P2	0.0232 (4)	0.0249 (4)	0.0310 (4)	−0.0009 (3)	0.0051 (3)	0.0006 (4)
Cl1	0.0347 (5)	0.0317 (4)	0.0342 (4)	0.0046 (3)	0.0031 (3)	0.0011 (3)

### Geometric parameters (Å, °)

**Table:**

C1—C2	1.389 (5)	C19—H19	0.9500
C1—C6	1.387 (5)	C20—C21	1.371 (6)
C1—P1	1.844 (3)	C20—H20	0.9500
C2—C3	1.388 (5)	C21—C22	1.390 (5)
C2—H2	0.9500	C21—H21	0.9500
C3—C4	1.389 (6)	C22—H22	0.9500
C3—H3	0.9500	C23—C24	1.385 (5)
C4—C5	1.367 (5)	C23—C28	1.399 (5)
C4—H4	0.9500	C23—P2	1.829 (3)
C5—C6	1.394 (5)	C24—C25	1.379 (5)
C5—H5	0.9500	C24—H24	0.9500
C6—H6	0.9500	C25—C26	1.374 (5)
C7—C12	1.387 (5)	C25—H25	0.9500
C7—C8	1.398 (5)	C26—C27	1.369 (5)
C7—P1	1.833 (3)	C26—H26	0.9500
C8—C9	1.383 (5)	C27—C28	1.384 (5)
C8—H8	0.9500	C27—H27	0.9500
C9—C10	1.381 (5)	C28—H28	0.9500
C9—H9	0.9500	C29—C37	1.416 (5)
C10—C11	1.376 (5)	C29—C33	1.427 (4)
C10—H10	0.9500	C29—C30	1.438 (5)
C11—C12	1.377 (5)	C29—Ru1	2.354 (3)
C11—H11	0.9500	C30—C31	1.424 (5)
C12—H12	0.9500	C30—Ru1	2.183 (3)
C13—C14	1.528 (5)	C30—H30	1.0000
C13—P1	1.848 (3)	C31—C32	1.417 (5)
C13—H13A	0.9900	C31—Ru1	2.165 (3)
C13—H13B	0.9900	C31—H31	1.0000
C14—C15	1.524 (5)	C32—C33	1.441 (5)
C14—H14A	0.9900	C32—Ru1	2.219 (3)
C14—H14B	0.9900	C32—H32	1.0000
C15—C16	1.530 (5)	C33—C34	1.414 (5)
C15—H15A	0.9900	C33—Ru1	2.352 (3)
C15—H15B	0.9900	C34—C35	1.365 (6)
C16—P2	1.836 (3)	C34—H34	0.9500
C16—H16A	0.9900	C35—C36	1.410 (6)
C16—H16B	0.9900	C35—H35	0.9500
C17—C22	1.389 (5)	C36—C37	1.363 (5)
C17—C18	1.390 (5)	C36—H36	0.9500
C17—P2	1.835 (3)	C37—H37	0.9500
C18—C19	1.377 (5)	Ru1—P1	2.2502 (9)
C18—H18	0.9500	Ru1—P2	2.2968 (8)
C19—C20	1.386 (6)	Ru1—Cl1	2.4467 (8)
			
C2—C1—C6	118.6 (3)	C25—C26—H26	119.9
C2—C1—P1	118.2 (2)	C26—C27—C28	120.1 (4)
C6—C1—P1	123.1 (3)	C26—C27—H27	119.9
C3—C2—C1	120.8 (3)	C28—C27—H27	119.9
C3—C2—H2	119.6	C27—C28—C23	120.5 (3)
C1—C2—H2	119.6	C27—C28—H28	119.7
C2—C3—C4	119.7 (4)	C23—C28—H28	119.7
C2—C3—H3	120.1	C37—C29—C33	119.4 (3)
C4—C3—H3	120.1	C37—C29—C30	133.2 (3)
C5—C4—C3	120.0 (3)	C33—C29—C30	107.4 (3)
C5—C4—H4	120.0	C37—C29—Ru1	128.2 (2)
C3—C4—H4	120.0	C33—C29—Ru1	72.28 (17)
C4—C5—C6	120.2 (4)	C30—C29—Ru1	65.16 (17)
C4—C5—H5	119.9	C31—C30—C29	107.6 (3)
C6—C5—H5	119.9	C31—C30—Ru1	70.21 (18)
C1—C6—C5	120.6 (3)	C29—C30—Ru1	78.12 (18)
C1—C6—H6	119.7	C31—C30—H30	125.8
C5—C6—H6	119.7	C29—C30—H30	125.8
C12—C7—C8	118.3 (3)	Ru1—C30—H30	125.8
C12—C7—P1	120.5 (3)	C32—C31—C30	109.2 (3)
C8—C7—P1	121.2 (3)	C32—C31—Ru1	73.2 (2)
C9—C8—C7	120.4 (3)	C30—C31—Ru1	71.56 (19)
C9—C8—H8	119.8	C32—C31—H31	125.3
C7—C8—H8	119.8	C30—C31—H31	125.3
C8—C9—C10	120.2 (3)	Ru1—C31—H31	125.3
C8—C9—H9	119.9	C31—C32—C33	107.0 (3)
C10—C9—H9	119.9	C31—C32—Ru1	69.11 (19)
C11—C10—C9	119.7 (3)	C33—C32—Ru1	76.74 (18)
C11—C10—H10	120.1	C31—C32—H32	126.2
C9—C10—H10	120.1	C33—C32—H32	126.2
C12—C11—C10	120.4 (4)	Ru1—C32—H32	126.2
C12—C11—H11	119.8	C34—C33—C29	119.9 (3)
C10—C11—H11	119.8	C34—C33—C32	131.6 (3)
C11—C12—C7	121.0 (3)	C29—C33—C32	108.5 (3)
C11—C12—H12	119.5	C34—C33—Ru1	127.2 (2)
C7—C12—H12	119.5	C29—C33—Ru1	72.41 (18)
C14—C13—P1	119.3 (2)	C32—C33—Ru1	66.65 (18)
C14—C13—H13A	107.5	C35—C34—C33	118.9 (4)
P1—C13—H13A	107.5	C35—C34—H34	120.5
C14—C13—H13B	107.5	C33—C34—H34	120.5
P1—C13—H13B	107.5	C34—C35—C36	121.3 (4)
H13A—C13—H13B	107.0	C34—C35—H35	119.4
C15—C14—C13	116.7 (3)	C36—C35—H35	119.4
C15—C14—H14A	108.1	C37—C36—C35	121.3 (4)
C13—C14—H14A	108.1	C37—C36—H36	119.3
C15—C14—H14B	108.1	C35—C36—H36	119.3
C13—C14—H14B	108.1	C36—C37—C29	119.2 (3)
H14A—C14—H14B	107.3	C36—C37—H37	120.4
C14—C15—C16	115.5 (3)	C29—C37—H37	120.4
C14—C15—H15A	108.4	C31—Ru1—C30	38.23 (12)
C16—C15—H15A	108.4	C31—Ru1—C32	37.69 (14)
C14—C15—H15B	108.4	C30—Ru1—C32	63.48 (13)
C16—C15—H15B	108.4	C31—Ru1—P1	89.21 (10)
H15A—C15—H15B	107.5	C30—Ru1—P1	114.21 (10)
C15—C16—P2	114.8 (2)	C32—Ru1—P1	101.18 (10)
C15—C16—H16A	108.6	C31—Ru1—P2	132.17 (10)
P2—C16—H16A	108.6	C30—Ru1—P2	99.27 (9)
C15—C16—H16B	108.6	C32—Ru1—P2	160.13 (9)
P2—C16—H16B	108.6	P1—Ru1—P2	94.88 (3)
H16A—C16—H16B	107.5	C31—Ru1—C33	60.97 (14)
C22—C17—C18	118.0 (3)	C30—Ru1—C33	61.09 (12)
C22—C17—P2	122.6 (3)	C32—Ru1—C33	36.61 (12)
C18—C17—P2	119.2 (3)	P1—Ru1—C33	137.29 (9)
C19—C18—C17	120.7 (4)	P2—Ru1—C33	127.67 (9)
C19—C18—H18	119.7	C31—Ru1—C29	61.28 (13)
C17—C18—H18	119.7	C30—Ru1—C29	36.72 (12)
C18—C19—C20	120.5 (4)	C32—Ru1—C29	61.13 (12)
C18—C19—H19	119.7	P1—Ru1—C29	149.38 (9)
C20—C19—H19	119.7	P2—Ru1—C29	99.26 (8)
C21—C20—C19	119.7 (4)	C33—Ru1—C29	35.31 (11)
C21—C20—H20	120.1	C31—Ru1—Cl1	137.05 (11)
C19—C20—H20	120.1	C30—Ru1—Cl1	154.00 (10)
C20—C21—C22	119.7 (4)	C32—Ru1—Cl1	101.22 (11)
C20—C21—H21	120.2	P1—Ru1—Cl1	88.51 (3)
C22—C21—H21	120.2	P2—Ru1—Cl1	90.75 (3)
C21—C22—C17	121.3 (3)	C33—Ru1—Cl1	93.83 (9)
C21—C22—H22	119.4	C29—Ru1—Cl1	118.14 (9)
C17—C22—H22	119.4	C7—P1—C1	102.34 (15)
C24—C23—C28	118.0 (3)	C7—P1—C13	103.63 (16)
C24—C23—P2	123.6 (3)	C1—P1—C13	99.75 (15)
C28—C23—P2	118.2 (2)	C7—P1—Ru1	118.56 (11)
C25—C24—C23	121.1 (3)	C1—P1—Ru1	109.17 (11)
C25—C24—H24	119.4	C13—P1—Ru1	120.43 (11)
C23—C24—H24	119.4	C23—P2—C16	102.80 (15)
C26—C25—C24	120.1 (4)	C23—P2—C17	100.73 (14)
C26—C25—H25	120.0	C16—P2—C17	101.01 (15)
C24—C25—H25	120.0	C23—P2—Ru1	116.19 (11)
C27—C26—C25	120.1 (3)	C16—P2—Ru1	120.06 (11)
C27—C26—H26	119.9	C17—P2—Ru1	113.29 (10)

## References

[bb1] Bruker (2005). *SADABS* Bruker AXS Inc., Madison, Wisconsin, USA.

[bb2] Farrugia, L. J. (1997). *J. Appl. Cryst.* **30**, 565.

[bb3] Farrugia, L. J. (1999). *J. Appl. Cryst.* **32**, 837–838.

[bb4] Franco, M., Giambattista, C., Angelo, S. & Massimo, M. (1989). *J. Organomet. Chem.* **370**, 305–318.

[bb5] Nonius (1998). *COLLECT.* Nonius BV, Delft, The Netherlands.

[bb6] Oro, L. A., Ciriano, M. A., Campo, M., Foces-Foces, C. & Cano, F. H. (1985). *J. Organomet. Chem.* **289**, 117–131.

[bb7] Otwinowski, Z. & Minor, W. (1997). *Methods in Enzymology*, Vol. 276, *Macromolecular Crystallography*, Part A, edited by C. W. Carter Jr & R. M. Sweet, pp. 307–326. New York: Academic Press.

[bb8] Sheldrick, G. M. (2008). *Acta Cryst.* A**64**, 112–122. 10.1107/S0108767307043930 18156677

[bb9] Tanase, T., Mochizuki, H., Sato, R. & Yamamoto, Y. (1994). *J. Organomet. Chem.* **466**, 233–236.

